# Artificial Intelligence Algorithms and Natural Language Processing for the Recognition of Syncope Patients on Emergency Department Medical Records

**DOI:** 10.3390/jcm8101677

**Published:** 2019-10-14

**Authors:** Franca Dipaola, Mauro Gatti, Veronica Pacetti, Anna Giulia Bottaccioli, Dana Shiffer, Maura Minonzio, Roberto Menè, Alessandro Giaj Levra, Monica Solbiati, Giorgio Costantino, Marco Anastasio, Elena Sini, Franca Barbic, Enrico Brunetta, Raffaello Furlan

**Affiliations:** 1 Internal Medicine, Humanitas Clinical and Research Center- IRCCS, 20089 Rozzano, Milan, Italy; dana.shiffer@humanitas.it (D.S.); maura.minonzio@humanitas.it (M.M.); alessandro.giajlevra@st.hunimed.eu (A.G.L.); franca.barbic@humanitas.it (F.B.); enrico.brunetta@humanitas.it (E.B.); raffaello.furlan@hunimed.eu (R.F.); 2 Department of Biomedical Sciences, Humanitas University, 20090 Pieve Emanuele, Milan, Italy; 3 IBM Italy, 20090 Segrate, Milan, Italy; MAURO_GATTI@it.ibm.com (M.G.);; 4 Centro Trombosi e Malattie Emorragiche, Humanitas Clinical and Research Center- IRCCS, 20089 Rozzano, Milan, Italy; v_pacetti@hotmail.com; 5 Faculty of Psychology, “Vita-Salute San Raffaele” University, 20132 Milan, Italy; annagiulia.bottaccioli@gmail.com; 6 Pronto Soccorso e Medicina D’Urgenza, Fondazione IRCCS Ca’ Granda Ospedale Maggiore Policlinico, Università degli Studi di Milano, 20122 Milan, Italy; monica.solbiati@gmail.com (M.S.); giorgic2@gmail.com (G.C.); 7 ICT Department, Humanitas Clinical and Research Center- IRCCS, 20089 Rozzano, Milan, Italy; marco.anastasio@humanitas.it; 8 GVM Care & Research, 48124 Ravenna, Italy; esini@gvmnet.it

**Keywords:** syncope, artificial intelligence, natural language processing, Emergency Department, electronic medical records

## Abstract

Background: Enrollment of large cohorts of syncope patients from administrative data is crucial for proper risk stratification but is limited by the enormous amount of time required for manual revision of medical records. Aim: To develop a Natural Language Processing (NLP) algorithm to automatically identify syncope from Emergency Department (ED) electronic medical records (EMRs). Methods: De-identified EMRs of all consecutive patients evaluated at Humanitas Research Hospital ED from 1 December 2013 to 31 March 2014 and from 1 December 2015 to 31 March 2016 were manually annotated to identify syncope. Records were combined in a single dataset and classified. The performance of combined multiple NLP feature selectors and classifiers was tested. Primary Outcomes: NLP algorithms’ accuracy, sensitivity, specificity, positive predictive value, negative predictive value, and F3 score. Results: 15,098 and 15,222 records from 2013 and 2015 datasets were analyzed. Syncope was present in 571 records. Normalized Gini Index feature selector combined with Support Vector Machines classifier obtained the best F3 value (84.0%), with 92.2% sensitivity and 47.4% positive predictive value. A 96% analysis time reduction was computed, compared with EMRs manual review. Conclusions: This artificial intelligence algorithm enabled the automatic identification of a large population of syncope patients using EMRs.

## 1. Introduction

Syncope is a common symptom encountered in clinical practice and may manifest itself in a wide spectrum of conditions ranging from benign (i.e., vasovagal syncope) to life-threatening disorders (i.e., sustained arrhythmias, acute myocardial infarction, pulmonary embolism, aortic dissection). In the latter group, the 7- to 10-day mortality risk is slightly lower than 1% [1], while the incidence of major adverse events at 30 days is about 4–5% [2]. The diagnosis of syncope is often a highly challenging exclusion-based process which is not supported by specific diagnostic tests. A quite variable percentage of patients, ranging from 13% to 83%, is usually admitted to the hospital because of diagnostic uncertainty and concern about the syncope underlying cause, without a significant increase in the diagnostic yield despite a high expenditure of economic resources [3]. 

Over the past 15 years, several syncope prediction tools were developed to guide clinician’s decision-making in the Emergency Department (ED) [4,5,6,7,8]. However, none of them proved to be superior to clinical judgement [3]. A possible explanation for this “predictive failure” may lie in the fact that, since short-term adverse events are rare in patients with syncope, robust statistical inferences can only be made after the recruitment of large cohorts of individuals enabling the inclusion of sufficient number of events. Thus, in the absence of reliable and automatic analysis of retrospective data, enormous time and resources would be required to accomplish that aim.

Previous attempts to automatically extract patients with syncope by means of the ICD codes alone lacked sensitivity, which means that a significant number of patients with syncope were lost [9,10]. The automated analysis of natural language might theoretically overcome this limitation because of its remarkable capability of extracting and classifying crucial features from a free text. Indeed, natural language processing (NLP) techniques were recently used to extract and classify relevant medical information from patients’ electronic clinical charts, including pediatric diseases [11] and bleeding among critically ill patients [12]. We hypothesized that this methodology might be suitable to automatically select large populations of syncope patients from clinical-administrative databases. The resulting large amount of data might promote future studies, enabling a more precise definition of the prognosis.

The aim of the present study was, therefore, to develop NLP algorithms for automatically identifying syncope episodes in ED medical records.

## 2. Materials and Methods

**Figure 1:**
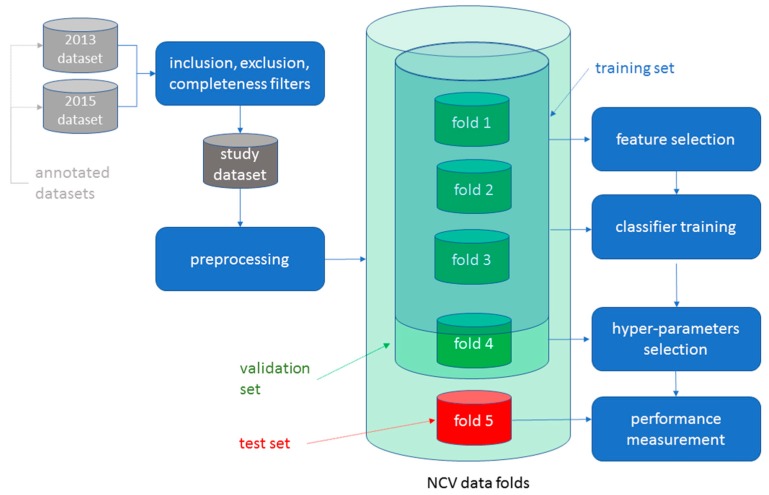
Data analysis methodology. The flow chart summarizes the data analysis methodology. The 2013 and 2015 datasets were obtained from the Humanitas Research Hospital electronic repository. Data were manually annotated by a group of six physicians. Manual annotation is the process of reviewing medical records to identify patients with syncope. Each dataset comprised 15,098 and 15,222 electronic medical records, respectively. After filtering for inclusion (i.e., age ≥ 18 years and data completeness) and exclusion (i.e., age < 18 years or data incompleteness) criteria, the two datasets were combined in a single study dataset which underwent a preprocessing based on cleaning and formatting operations. The final study dataset was analyzed using a five-folds Nested Cross Validation (NCV). This latter was based on multiple iterations in each of which data was randomly split in five folds, i.e., five subsets of electronic medical records of equal magnitude. Four folds were used for feature selection/training/validation, whereas fold five was used for testing. Feature selection recognized the grams that were most relevant for identifying patients with syncope. Classifier training identified the model parameters that best represented the dataset. Validation enabled the identification of the best hyper-parameters model. NCV allowed the use of the entire set of data for either the training, validation or testing procedures, thus achieving algorithm optimal performance.

This is a retrospective diagnostic study. The data analysis methodology we have used consists of the following phases: 1. Data acquisition; 2. Data labelling; 3. Data preprocessing; 4. Feature selection and extraction; 5. Classifiers and hyper-parameters selection; and 6. Model training and evaluation (Figure 1). This methodology is similar to the one described by Mirończuk and Protasiewicz [13]. The NLP algorithms were built in phases 3, 4 and 5.

### 2.1. Data Acquisition and Study Population

Electronic Medical Records (EMRs) of all consecutive patients older than 18 years, evaluated at Humanitas Research Hospital ED from 1 December 2013 to 31 March 2014 (2013 dataset) and from 1 December 2015 to 31 March 2016 (2015 dataset), were analyzed (Figure 1). EMRs of patients younger than 18 years of age were excluded. Humanitas Research Hospital is a tertiary academic center in the Milan area with an ED which provides care for more than 50,000 patients per year. From both datasets, the following fields were taken into consideration: (a) International Classification of Disease, 9th revision, Clinical Modified (ICD-9-CM) coding at ED discharge; (b) patient clinical features as described by triage operators; (c) ED physician’s notes of patient’s medical history; and (d) discharge diagnosis from the ED. All these fields, but the ICD-9 code, contained unstructured text written in Italian.

Each dataset was extracted from the Humanitas Research Hospital electronic repository by ICT Department experts and was made available in the form of a Microsoft Excel spreadsheet. Each ED patient encounter corresponds to one or multiple records in the spreadsheet depending on the number of ICD-9 codes assigned to the encounter (i.e., each record corresponds to a single ICD-9 code).

The study was approved by Humanitas Research Hospital Ethics Committee on Human Research, in compliance with the current Italian privacy regulations. 

### 2.2. Data Labelling

Data labelling (or annotation) is the process of manually reviewing data to add information that will be used to train and validate the classification algorithms. In our study, manual evaluation and annotation of de-identified datasets were performed by a group of six physicians (F.D., V.P., A.G.B., D.S., M.M., F.B.), hereafter mentioned as “annotators”. Each record was annotated as positive or negative for the presence of syncope by a single operator. Syncope was considered present by annotators if either the term “syncope” was clearly reported in the ED discharge diagnosis and/or medical history description or if the description of the episode agreed with the European Society of Cardiology guidelines definition of syncope [14]. Clinical judgment of emergency physicians was considered the reference standard for syncope diagnosis. NLP-based classification was subsequently compared with annotators classification. To assess the consistency of doctors’ annotations, we computed the kappa interrater reliability test.

Physicians that annotated the records did not take part in the development of algorithms and vice-versa the developers of algorithms did not contribute to the annotation. 

### 2.3. Data Preprocessing

The data preprocessing consisted of a long series of data cleaning and formatting operations (Figure 1). Moreover, in this phase, an explorative analysis of the data was conducted in order to frame the rest of the study. 

### 2.4. Feature Selection

Feature selection is the process of identifying those characteristics that are deemed to be relevant for a specific classification problem. For instance, the presence of a word like “syncope” or “fall” in the record is a characteristic that may identify patients with syncope. These characteristics can be used to transform textual data into numbers by assigning the number one to all records that contain the selected words and zero to all the others. These numeric vectors are subsequently used by classification algorithms for training and prediction (Figure 1).

In the selection of syncope features, we used two main approaches: (a) a manual selection, based on expert clinical judgement, and (b) an automatic selection, based on probabilistic criteria.

*Manual selection* of features consisted in the identification of n-grams (i.e., n adjacent words) that can better differentiate patients with syncope from patients without syncope by means of a two-step process. First, unigrams, bigrams and trigrams that occurred most often in the records’ free text fields were extracted to build an initial list of terms. Second, this list was reviewed by the annotators that, according to their expert clinical judgement, filtered out terms that were expected not to enable the discrimination of patients (e.g., the unigram “disease”). Unigrams were subsequently stemmed to overcome potential inflection variants (e.g., “fall” and “fallen”). The list of n-grams selected is provided in Table 1. Similarly, the manually selected feature for the ICD-9 field identifying patients with syncope was settled as “780.2”. The feature selector based on this list of manually selected terms was subsequently called “HuMan”.

*Automatic selection* was performed through several classical term selection algorithms: Gini Index (GI), Normalized Gini Index (NGI), Information Gain, and Chi Squared [15,16]. Indices were computed on unigrams, bigrams and trigrams after filtering out low frequency and high frequency terms. Both frequency thresholds and term selection algorithms were chosen in order to maximize F3 performance of the classification algorithms on the validation dataset (see below). Finally, we decided to use NGI that exhibited superior F3 performance. GI was also used to identify the ICD-9-CM codes.

### 2.5. Classifiers and Hyper-Parameters Selection

A classifier is an algorithm in which the objective is to predict the value of a categorical variable. In machine learning methodology, classifier algorithms are typically implementations of functions with a known structure (e.g., a logistic function) but in which values depend on unknown parameters that must be learned from data feeding. 

Different classification algorithms have been used to classify each record as positive or negative for the presence of syncope according to the extracted features. Classifiers that have been used include: Naïve Bayes (NB, Bernoulli), Support Vector Machines (SVM, Linear), Decision Trees, and MLP Neural Networks. Performance of the classification algorithms was measured through the F3 parameter. F3 is a metric that takes into account sensitivity and positive predictive value (PPV), giving triple importance to the former rather than to the latter. F3 was chosen by the investigators because it has been deemed more important not to miss patients with syncope rather than erroneously include patients without syncope. 

Notably, the performance of a classification algorithm is highly dependent on hyper-parameters. In this context, a hyper-parameter is a parameter whose value is set before training of the algorithm to an initial value and subsequently tuned by searching through a pre-defined search space of possible values for those values that maximize F3 performance on a validation set. Hyper-parameter tuning and overall performance computation was performed through a five-fold Nested Cross Validation (NCV) [17], see Figure 1. 

In our case, we had two main sets of hyper-parameters: those used to tune the feature selector algorithms, and those used to tune the classifiers. Examples of (automatic) feature selector hyper-parameters we included in our search space were, for instance, the number of unigrams (respectively bigrams, trigrams) for each of the main three textual data fields (patient clinical features as described by triage operators, ED physician’s notes of patient’s medical history and discharge diagnosis from the ED). Examples of classifier hyper-parameters were the under-sampling ratio (for Bernoulli classifiers) and the class-weight for SVM. 

In each iteration of the inner cross validation of the NCV algorithm, a sequence of hyper-parameter values was selected from the search space. These values were used by feature selectors to identify the relevant features to be used by the classification algorithms in that specific iteration and by classifiers to compute the iteration F3 score on the validation fold. The iteration F3 scores were subsequently compared to obtain the optimal score to be used to compute the overall performance on the test fold. 

### 2.6. Model Training and Evaluation

The performance of classification algorithms in correctly discriminating patients with syncope from patients without syncope was initially evaluated by using the 2013 dataset as a training set and the 2015 dataset as a validation/test set (Figure 1). Subsequently, the two datasets were merged, and the NCV methodology was used, thereby achieving the double benefit of increasing the training set size and using all the data items for both training and validation. Notably, a large training set size is useful to improve algorithms performance, while using all data may help to increase algorithm generalizability (Figure 1).

Different algorithm performances were compared using a Welch Two Sample t-test. A *p* value < 0.05 was considered significant. Based on the results of the NCV, we only kept NB and SVM that were characterized by a greater performance. In addition, the core phases feature selection, classifier and hyper-parameters selection and model training and evaluation were iterated several times with the purpose of testing multiple combinations, thereby identifying those delivering the best performance.

NLP algorithms were developed using only open source tools to foster reproducibility (Python 3.6.8, NLTK 3.4, scikit-learn 0.20.2, R 3.3.1). All statistical analyses were conducted using R 3.3.1. 

## 3. Results

We manually analyzed 15,098 EMRs from the 2013 dataset (251 syncope episodes) and 15,222 from the 2015 dataset (320 syncope episodes). Kappa interrater reliability was 93.7%; it was measured on 96 randomly extracted EMRs, independently and blindly annotated by two operators. 

The patients’ demographic and clinical characteristics, obtained from the analyzed charts, are summarized in Table 2.

Table 3 shows the performance of the algorithms that resulted in the best F3 value. In particular, the model consisting of the selector NGI and the classifier SVM reported an F3 value of 84.0%, corresponding to 92.2% sensitivity and 47.4% PPV. 

SVM classifier performed significantly better than NB used in combination with the “HuMan” manual selector (F3 value, 78.8% vs. 68.1%; difference, 10.7%; 95%CI, −14.2–7.2; *p* < 0.001). NGI feature selector performed significantly better than “HuMan” selector when used in combination with the best SVM classifier (F3 value, 84.0% vs. 78.8%; difference, 5.2%; 95%CI, −8.3–2.1; *p* = 0.005) (Table 4).

We calculated that to theoretically achieve 100% sensitivity, which means not missing a single syncope patient, a 221 days/person time would have been required to manually analyze every single medical record out of the total 30,320. In contrast, the automatic analysis using our algorithm would enable the identification of syncope patients (“true positives”) by analyzing only 4% of medical records, with an expected analysis time of 8.8 days/person.

## 4. Discussion

The results of the present study suggest that an algorithm based on the SVM as classifier and the NGI as feature selector enabled us to identify patients with syncope from EMRs with high sensitivity and acceptable PPV, resulting in a 96% reduction in the work-time needed for manual identification. Notably, the algorithm with a greater F3 value allowed us not to miss the largest number of syncopal episodes.

To our knowledge, this is the first attempt to apply NLP for the recognition of patients with syncope in the ED. Taggart et al. [12] compared two NLP methods for the identification of bleeding among critically ill patients through the analysis of EMRs. In their analysis, based on 1650 notes taken from the MIMIC database, the Rule-Based approach showed excellent performances (62.7% PPV and a 97.1% NPV) in identifying the 146 notes related to a bleeding event. Recently, Liang et al. [11] developed a NLP-based diagnosis support system that enabled the identification of the most common pediatric diseases from EMRs. Based on 6183 charts, their algorithm classified pediatric patients into pre-specified diagnostic categories with an accuracy comparable to junior physicians. Our algorithm attained comparable classification performances but for a much less frequent event, as syncope accounts for about 1–3% of ED visits [14]. The rareness of the event of interest accounts for the need of a much larger number of charts that had to be annotated in the current study. Taken together, these applications of artificial intelligence (AI) solutions are effective examples of how NLP algorithms may be of help in exploiting the huge amount of unstructured clinical data contained in EMRs repositories.

A cardinal strength of our algorithm is its generalizability, both in terms of languages it can be applied to and in terms of the event it can seek for. Indeed, although in the present study NLP has only been used on Italian EMRs, the dependence on this language is only related to the stemming algorithms and the annotated data. Thus, should our algorithm be trained on annotated data using a different language, performances similar to those achieved in the present study are expected. Moreover, the use of a different language characterized by a richer NLP tool set, such as English, is likely to be associated with greater performances. This is due to the availability of clinical Named Entity Recognition tools, like the UMLS Metamap [18], that may improve the identification of medical concepts into a text. Similar considerations apply to the use of techniques like word embedding [19]. 

Likewise, at no point is the algorithm depending on the specific problem of syncope identification. It is only through the annotation and training process that the trained model becomes dependent on this specific objective. It is therefore reasonable to hypothesize that the present algorithm could be useful for the identification of patients with other medical conditions such as dyspnea or different diseases. 

Syncope is a frequent cause of ED presentation and hospital admission [3]. It is a common symptom of numerous conditions, and its prognosis is highly heterogeneous. Despite numerous attempts, no risk stratification tools superior to clinical judgement have been developed so far [3]. This may be due to the fact that related short-term adverse events are rather rare, being about 1% at 10 days from the index episode [1]. Thus, in order to include a significant number of events leading to robust prognostic conclusions, a prospective enrollment of large cohorts of patients is needed. This procedure entails considerable use of time and resources. AI techniques based on NLP might overcome these constraints by retrieving data through the analysis of EMRs that are continuously generated during daily clinical activities and stored in hospital ad hoc electronic repositories.

In addition, the creation of large syncope patient datasets could enable the set-up of more accurate automatic risk prediction tools. Indeed, according to the latest ESC guidelines for syncope management [14], there are more than 40 potential prognostic factors, many of which are rare. Therefore, to automatically identify these factors from ED physicians’ unstructured notes, NLP algorithms become strictly dependent on the availability of a large amount of data, a crucial point that was achieved by the current study. Moreover, the automatic identification of prognostic factors of syncope, jointly with an inference engine that acts accordingly to the over 100 rules contained in the clinical guidelines, paves the way for developing a software that could be used in ED to ensure full compliance with guidelines, thereby reducing variability in clinical management and, ultimately, the number of mistreated syncope patients.

A step beyond the sheer application of guidelines may be the development of algorithms allowing a better prediction of patients’ risk. Artificial neural networks (ANNs) were previously prospectively tested in ED syncope risk stratification [18], reporting a predictive accuracy comparable to that of the currently available prognostic tools. However, the used methodology was constrained by data availability, size and characteristics and, therefore, would have benefitted from leveraging administrative data containing a huge amount of untapped information. In addition, ANNs predicted syncope patients’ hospitalization with 100% sensitivity and 79% specificity [19], thus potentially increasing the appropriateness of medical treatment and, consequently, hospital efficiency. However, since no external validation of these models was performed, their generalizability still needs to be confirmed. 

## 5. Limitations

Some limitations of the current study should be acknowledged. 

First, the algorithm we set up was derived and tested using EMRs from a single institution ED. Therefore, it is possible that our results depend partially on medical jargon used at Humanitas Research Hospital. Algorithm performance needs an external validation using different patients’ settings and possibly various institutions. 

Second, manual annotation and natural language analysis were performed on free texts in Italian; therefore, our results may not be completely reproducible in languages other than Italian. Again, this point should be addressed by an external validation procedure using different datasets. It should be pointed out that, in addition to the manual selector (“HuMan”), we worked with broadly available feature selection algorithms, so that we are confident that our results may be easily reproduced by other researchers using different languages. 

Finally, we decided to compare our best algorithm performance with the clinical judgment of the emergency physician. This could have modified the diagnostic accuracy of our algorithm should the syncope be misdiagnosed. However, since syncope diagnosis is often based on exclusion without any specific test as the diagnostic support, the physician clinical judgment remained the only reference we considered as highly appropriate to test the algorithm effectiveness.

## 6. Conclusions

The use of the present NLP-based algorithm might result in significant reduction in the required time to analyze large databases with low costs and high reproducibility, in the Italian language. However, external validation on a different patient population is necessary to confirm and strengthen these results. 

The capability to automatically select large study populations might provide a huge amount of data suitable for prompt analysis and possibly real time prognostic stratification. Therefore, individual clinical and demographic features of a new patient presenting to the ED might be compared with those characterizing a subset of similar individuals of known outcome, thus leading to an effective personalization of the prognosis and improvement of syncope management by the physician in charge. 

## Figures and Tables

**Table 1:** N-grams most frequently associated with the presence of syncope.

	Humanitas Syncope n-Grams
Unigrams	‘assenza’ (‘absence’), ‘caduta’ (‘fall’), ‘capogiro’ (‘dizziness’), ‘clonie’ (‘clonus’), ‘ipotensione’ (‘hypotension’), ‘lipotimia’ (‘lipotimia’), ‘malessere’ (‘malaise’), ‘malore’ (‘illness’), ‘prelipotimia’ (‘prelipotimia’), ‘presincope’ (‘presyncope’), ‘prodromi’ (‘prodromes’), ‘sincope’ (‘syncope’), ‘svenimento’ (‘faint’), ‘trauma’ (‘trauma’), ‘trovato’ (‘found’), ‘vertigini’ (‘dizziness’)
Bigrams	‘crisi epilettica’ (‘epileptic crisis’)
Trigrams	‘ferita lacero contusa’ (‘lacerated bruised wound’), ‘perdita di coscienza’ (‘loss of consciousness’)

**Table 2:** Patient characteristics of the 2013 and 2015 datasets.

Characteristic	2013 Dataset	2015 Dataset	Total
Electronical medical records, No.	15,098	15,222	30,320
Unique patients, No.	12,535	12,831	25,366
Female, No. (%)	6303 (50.3)	6445 (50.2)	12,748 (50.2)
Age, mean (SD), y	54.6 (20.0)	56.0 (20.0)	55.3 (20.0)
Syncope present, No. (%)	251 (1.7)	320 (2.1)	571 (1.9)
Reason for ED admission *			
Abdominal pain	1051 (7.0)	886 (5.8)	1937 (6.4)
Chest pain	557 (3.7)	661 (4.3)	1218 (4.0)
Lumbar pain	455 (3.0)	432 (2.8)	887 (2.9)
Cervicalgia	406 (2.7)	266 (1.7)	672 (2.2)
Fatigue and malaise	249 (1.6)	380 (2.5)	629 (2.1)
Renal colic	281 (1.9)	265 (1.7)	546 (1.8)
Primary hypertension	203 (1.3)	296 (1.9)	499 (1.6)
Cerebrovascular disease	192 (1.3)	296 (1.9)	488 (1.6)
Heart failure	187 (1.2)	174 (1.1)	361 (1.2)
Coronary heart disease	132 (0.9)	157 (1.0)	289 (1.0)

* Reasons for ED admission as reported by ICD-9 code at ED discharge.

**Table 3:** Algorithms performance for identifying syncope from EMRs.

Algorithm	Accuracy	Sensitivity	PPV	F3 Score ^a^	NPV	Specificity
NB-HuMan	98.3	70.6	51.8	68.1	99.5	98.8
SVM-HuMan	96.7	92.6	34.1	78.8	99.9	96.8
NB-NGI	98.0	89.3	47.2	82.0	99.8	98.1
SVM-NGI	98.0	92.2	47.4	84.0	99.9	98.1

Abbreviations: NB, Naïve Bayes; HuMan, Humanitas Manual; SVM, Support Vector Machines; NGI, Normalized Gini Index. PPV, positive predictive value; NPV, negative predictive value. ^a^ F score is defined as the harmonic mean of the sensitivity and positive predictive value. F3 gives triple importance to sensitivity over precision (average value).

**Table 4:** Algorithms performance comparison.

Algorithm 1	Algorithm 2	Algorithm 1 F3 Score	Algorithm 2 F3 Score	95% CI	*p* Value
NB-HuMan	SVM-HuMan	68.1	78.8	−14.2–7.2	<0.001
SVM-HuMan	SVM-NGI	78.8	84.0	−8.3–2.1	0.005

Abbreviations: NB, Naïve Bayes; HuMan, Humanitas Manual; Support Vector Machines, SVM; Normalized Gini Index, NGI. F score is defined as in Table 2.
